# Update follikuläres Schilddrüsenkarzinom – Was ist für Chirurg*innen relevant?

**DOI:** 10.1007/s00104-025-02276-1

**Published:** 2025-03-27

**Authors:** Nicolas Schlegel

**Affiliations:** Klinik und Poliklinik für Allgemein‑, Viszeral‑, Transplantations‑, Gefäß- und Kinderchirurgie, Universitätsklinik Würzburg, Oberdürrbacher Straße 6, 97080 Würzburg, Deutschland

**Keywords:** Klassifikation, Präoperative Diagnostik, Prognose, Resektionsausmaß, Chirurgisches Vorgehen, Classification, Preoperative diagnostics, Prognosis, Surgical procedure, Extent of resection

## Abstract

Das follikuläre Schilddrüsenkarzinom (FTC) ist die zweithäufigste Form des differenzierten Schilddrüsenkarzinoms und macht etwa 5–15 % aller Schilddrüsenmalignome aus. Das FTC wird gemäß der WHO(World Health Organization)-Klassifikation von 2022 in drei Subtypen gegliedert, die sich in Gesamtprognose und Rezidivwahrscheinlichkeit deutlich unterscheiden. Darüber hinaus ist die Kenntnis gutartiger follikulärer Tumoren und Low-grade-Neoplasien wichtig. Prognostische Faktoren bei den malignen Veränderungen sind neben dem Lebensalter, die Größe des Tumors, invasives Wachstum, das Vorliegen von Angioinvasionen sowie das Vorhandensein von Fernmetastasen. Die chirurgische Vorgehensweise orientiert sich an diesen Faktoren. Entsprechend unterscheidet sie sich zwischen den Subtypen des FTC. Eine genaue Kenntnis der einzelnen Subtypen von Schilddrüsentumoren ist erforderlich, um eine individuell angemessene chirurgische Therapieentscheidung treffen zu können.

## Abstract (en)

Follicular thyroid cancer (FTC) is the second most frequent form of differentiated thyroid cancer, accounting for approximately 5–15% of all thyroid malignancies. According to the World Health Organization (WHO) classification from 2022 FTC is divided into three subtypes, which clearly differ in the overall prognosis and probability of recurrence. Furthermore, knowledge about benign follicular tumors and low-grade neoplasms is important. Prognostic factors for malignant alterations include patient age, tumor size, invasive growth, the presence of angioinvasion and the occurrence of distant metastases. The surgical management is guided by these factors and therefore varies between the different FTC subtypes. In summary, a precise understanding of the individual subtypes of thyroid tumors is essential to be able to make an appropriate and personalized decision on surgical treatment.

Das follikuläre Schilddrüsenkarzinom (follikuläres Karzinom der Schilddrüse, FTC) ist die zweithäufigste Form des differenzierten Schilddrüsenkarzinoms und macht etwa 5–15 % aller Schilddrüsenmalignome aus. Es tritt bevorzugt in Regionen mit Jodmangel auf. Frauen sind etwa 5‑mal häufiger betroffen als Männer. Der Erkrankungsgipfel liegt um das 50. Lebensjahr und ist damit deutlich später als beim papillären Schilddrüsenkarzinom (PTC). Im Vergleich zum PTC zeigt das FTC eine höhere Neigung zur hämatogenen Metastasierung. Die Prognose ist mit einer 10-Jahres-Gesamtüberlebensrate von über 90 % sehr gut.

## Hintergrund

Das FTC ist ein bösartiger Tumor der Schilddrüse, der aus den follikulären Epithelzellen hervorgeht. Schlüsseleigenschaften, die zur Diagnose eines FTC führen, sind neben dem Wachstumsverhalten die Kapselinvasion und die Angioinvasion. Diese haben zu einer Einteilung in aktuell drei Subtypen geführt:

1. minimal-invasives follikuläres Schilddrüsenkarzinom (MIFTC ohne Kapselinvasion),
2. angioinvasives follikuläres Schilddrüsenkarzinom (MIFTC mit Angioinvasion),
3. breitinvasives follikuläres Schilddrüsenkarzinom (WIFTC).

Die vormals onkozytäre Form des follikulären Schilddrüsenkarzinoms wird gemäß der Klassifikation der World Health Organization (WHO) von 2022 als eigene Entität, nämlich als onkozytäres Schilddrüsenkarzinom (OTC) aufgeführt.

Bei den drei Entitäten des FTC ist das 40-monatige krankheitsfreie Überleben deutlich unterschiedlich mit 97 % beim minimal-invasiven FTC, 81 % beim angioinvasiven FTC und bei 46 % beim breitinvasiven FTC [21]. Hier ist für das Verständnis wichtig zu erwähnen, dass minimal-invasive FTC Angioinvasionen zeigen können und angioinvasive (häufig auch als enkapsulierte bezeichnet) FTC auch Kapseldurchbrüche aufweisen können. Übergreifend über alle Entitäten liegt das 5‑ und 10-Jahres-Gesamtüberleben beim FTC über 90 % [20]. Die Prognose des onkozytären Schilddrüsenkarzinom wird in Bezug auf das krankheitsfreie Überleben und den Anteil an radiojodtherapierefraktären Verläufen als zwischen den minimal-invasiven und breitinvasiven Varianten des FTC liegend eingestuft [25]. Insgesamt wurde das 5‑Jahres-Überleben beim OCA in Abhängigkeit des Vorhandenseins von Fernmetastasen zwischen 85–91 % angegeben [4].

## WHO-Klassifikation von 2022 führt zu neuen Subklassifikationen

Die Weltgesundheitsorganisation (WHO) hat in ihrer Klassifikation von 2022 einen differenzierteren Ansatz zur Diagnostik von Schilddrüsentumoren follikulären Ursprungs festgelegt (Tab. 1; [4]). Dieser erhöht zum einen die Komplexität, trägt aber auf der anderen Seite zu einer besseren Einschätzung der Prognose im Einzelfall bei. Auch wenn für die neueren Entitäten keine belastbaren Daten für das exakte chirurgische Vorgehen existieren, ist es für endokrine Chirurgen wichtig, diese zu kennen, um an deren Risikoprofilen interdisziplinär individualisierte Vorgehensweisen diskutieren zu können.

**Tab. 1:** Eine Übersicht über die WHO-Klassifikation 2022 von Schilddrüsentumoren follikulären Ursprungs. (Mod. nach [4])

Gutartige Tumoren	Follikuläre noduläre Erkrankung („Knotenstruma“)
	Follikuläres Adenom
	Follikuläres Adenom mit papillärer Architektur
	Onkozytäres Adenom
Low-risk-Neoplasien	Nichtinvasive follikuläre Schilddrüsenneoplasie mit papillären Kerneigenschaften (NIFTP)
	Schilddrüsentumor mit unsicherem malignem Potenzial
	Hyalisierender trabekulärer Tumor
Maligne Neoplasien	Follikuläres Schilddrüsenkarzinom
	Invasive enkapsulierte follikuläre Variante des papillären Schilddrüsenkarzinoms
	Papilläres Schilddrüsenkarzinom
	Onkozytäres Schilddrüsenkarzinom
	*Schilddrüsenkarzinom aus Zellen follikulären Ursprungs* Hochdifferenziertes High-grade-Schilddrüsenkarzinom Niedrig differenziertes Schilddrüsenkarzinom
	Anaplastisches Schilddrüsenkarzinom aus Zellen follikulären Ursprungs

^a^Nicht aufgeführt sind Schilddrüsenkarzinome nichtfollikulären Ursprungs wie das medulläre Schilddrüsenkarzinom

**Abb. 1:**
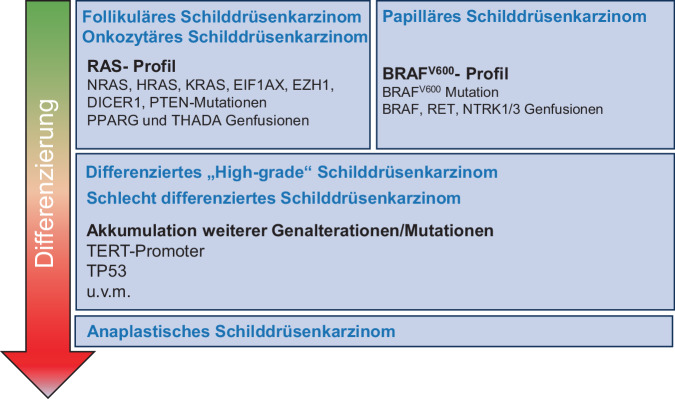
Stark vereinfachtes molekulares Modell der Tumorinitiation und -progression bei Schilddrüsenkarzinomen follikulären Ursprungs. Während sich die gut differenzierten Schilddrüsenkarzinome anhand ihres molekularen Profils gut unterscheiden lassen, wird dies bei schlechterer Differenzierung schwieriger, da hier eine vermehrte und heterogene Genalteration und eine höhere Mutationslast vorliegen. Der *Pfeil* links schematisiert den Übergang von gut differenziert (*grün*) zu nicht differenzierten (*rot*) Entitäten. (Die Abbildung wurde stark vereinfacht und modifiziert nach [15])

**Abb. 2:**
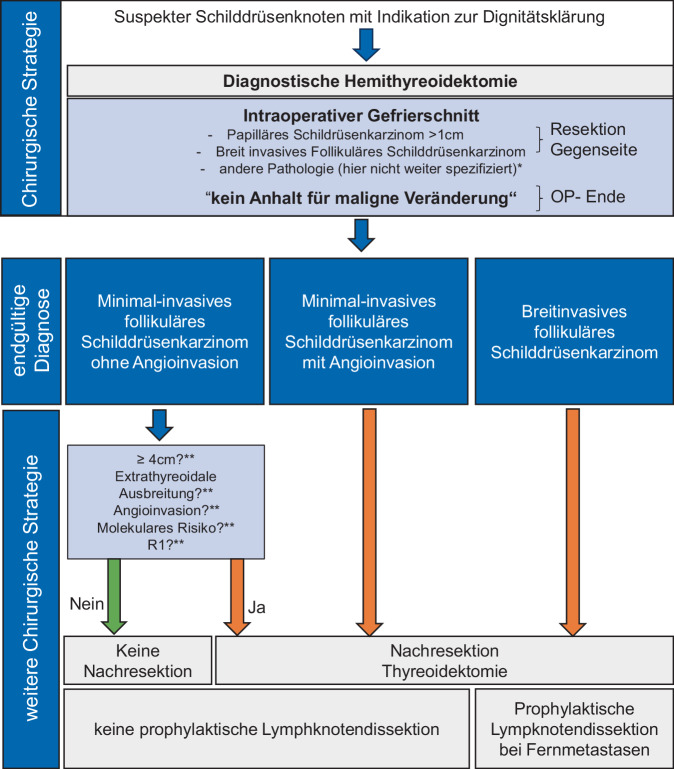
Würzburger Algorithmus zum chirurgischen Vorgehen bei follikulären Schilddrüsenkarzinomen. Basierend auf den national und international publizierten Leitlinien wird das dargestellte Schema zur Abklärung und chirurgischen Therapie follikulärer Schilddrüsenkarzinome verfolgt. *Stern* Zur Vereinfachung der Abbildung sind hier andere Pathologien und die chirurgische Vorgehensweise nicht genannt; *Doppelstern* Hier erscheint das Vorliegen einer oder mehrerer dieser prognostischen Faktoren ausreichend, um die Nachresektion im Sinne einer Thyreoidektomie zu rechtfertigen. Der Patient/die Patientin sollte in diese Entscheidung mit einbezogen werden. Nicht aufgeführt ist, dass wir beim Nachweis von Lymphknotenmetastasen eine befallsorientierte Kompartmentresektion durchführen. *OP* Operation

Insgesamt wird in der WHO-Klassifikation von 2022 den molekularen Veränderungen und deren inzwischen bekannter prognostischer Bedeutung vermehrt Rechnung getragen. Diese sind in einem stark vereinfachten Modell in Abb. 1 dargestellt. Auf dieser Basis wurde ein 3‑stufiger Algorithmus zur pathologischen Diagnostik und Einordnung von Schilddrüsentumoren vorgeschlagen [6]. Hiernach erfolgt die erste Einordnung des Schilddrüsentumors nach dessen zellulärem Ursprung (follikulär vs. medullär). Im nächsten Schritt erfolgt die Charakterisierung des molekularen Profils und dann die Einordnung gemäß klassischer histologisch-morphologischer Eigenschaften. Da letztere sehr eng mit molekularen Veränderungen korrelieren, spielt in der täglichen Praxis weiterhin die postoperative histomorphologische Einordnung der Schilddrüsentumoren die wichtigste Rolle. Die molekulare Diagnostik wird vor allem zur Ergänzung eingesetzt, vor allem dann, wenn die histomorphologische Einordnung nicht eindeutig ist. Da diese genaue Einordnung erst postoperativ stattfinden kann, spielt dies bei der Planung des chirurgischen Vorgehens vor dem Ersteingriff eine untergeordnete Rolle (Abb. 2).

## Präoperative Diagnostik – gibt es neue Entwicklungen?

Nach wie vor bleibt es unmöglich, das FTC präoperativ sicher zu diagnostizieren oder auszuschließen. Anhand morphologischer Kriterien ist die Feinnadelzyologie nicht in der Lage, ein FTC von einer benignen Läsion zu unterscheiden. Es besteht bei höhergradigen Bethesda-Kategorien (III–V) die Option einer detaillierten molekularen Aufarbeitung [1]. In prospektiven Studien konnte damit in 50–60 % der untersuchten Knoten eine Mutation ausgeschlossen werden, die auf eine maligne Transformation hinweist [17, 27]. Da jedoch das Fehlen genetischer Alterationen keine Benignität beweist, kann eine grundsätzliche Empfehlung zur molekularpathologischen Analyse des Materials der Schilddrüsenfeinnadelbiopsie bisher nicht ausgesprochen werden. Für die molekularpathologische Analyse des Schilddrüsenfeinnadelpunktats – die in Deutschland bislang kein Standardverfahren ist – stehen derzeit im Wesentlichen die diagnostischen Marker *BRAF-*^*V600E*^-Mutation, *RAS*-Mutationen, *RET*- oder *NTRK*-Fusionen zur Verfügung (Abb. 1). Aufgrund der oben beschriebenen Überlappungen und Heterogenität molekularer Veränderungen bleibt aber trotzdem eine Restunsicherheit, die in meisten Fällen trotz aufwendiger molekularer Untersuchungen zur klärenden, diagnostischen Hemithyreoidektomie führen wird.

Die Sonographie mit sorgfältiger Beurteilung der Knoten auf Basis der TIRADS-Kriterien bleibt essenziell

Unabhängig davon bleibt es dabei, dass eine sonographische Untersuchung mit sorgfältiger Beurteilung der Knoten auf Basis des Thyroid Imaging Reporting and Data System (TIRADS) essenziell ist. Ein eindeutiger Vorteil im Vergleich verschiedener Systeme scheint nicht vorhanden zu sein [24]. In einigen Aspekten ist das American College of Radiology (ACR)-TIRADS-System vorteilhaft [28].

Vielfach diskutiert wurde in den letzten Jahren die Durchführung einer ^99m^Tc-MIBI(Methoxyisobutylisonitril)-Szintigraphie zur Einschätzung der Dignität hypofunktioneller Schilddrüsenknoten. Der Wert dieser Untersuchung liegt hierbei vor allem in ihrem hohen negativen prädiktiven Wert von über 90 %. Eine spezifische Aussage bez. des Vorliegens maligner Veränderungen ist mit dieser Untersuchungsmethode nicht möglich, sodass diese nicht zum Screening von Schilddrüsenknoten eingesetzt werden sollte [30].

Vergleichbares gilt für die ^18F^FDG-PET(Fluorodeoxyglucose-Positronenemissionstomographie)-Untersuchung, die einen hohen negativen prädiktiven Wert von 93 % zum Ausschluss eines malignen Wachstums aufweist, aber mit einer Sensitivität von 89 % und Spezifität von 55 % nur einen positiv prädiktiven Wert von 41 % aufweist. Damit ist auch diese Untersuchung für ein präoperatives Screening zur Einschätzung der Dignität von Schilddrüsenknoten nicht zu empfehlen [8, 29].

## Bedeutung des intraoperativen Gefrierschnitts beim FTC

Die Durchführung eines intraoperativen Gefrierschnitts (Schnellschnitts) ist sinnvoll, wenn durch das zu erwartende Ergebnis eine chirurgische Konsequenz erfolgen kann. Grundsätzlich sollte bei präoperativem Verdacht auf einen malignen Schilddrüsenknoten ein intraoperativer Gefrierschnitt zur Dignitätsklärung durchgeführt werden. Auch wenn das minimal-invasive und das angioinvasive FTC in der Regel nicht im Schnellschnitt erkannt werden können, so gelingt es doch in vielen Fällen zumindest das breitinvasive FTC zu diagnostizieren [2, 19]. Obwohl dieses insgesamt selten ist und letzteres kontrovers diskutiert wird, führen wir im Zentrum auch bei präoperativ als „follikuläre Neoplasie“ klassifizierten Knoten immer intraoperative Schnellschnitte durch, um durch die mögliche frühe Erkennung von Pathologien, die Rate an Komplettierungsoperationen soweit wie möglich zu reduzieren.

Bei feinnadelzytologisch „malignomverdächtigem Punktat“ oder bei „sicherer Malignität“ ist der intraoperative Gefrierschnitt sinnvoll, um ggf. eine NIFTP („noninvasive follicular thyroid neoplasm with papillary-like bnuclear features“) vom PTC bereits frühzeitig unterscheiden zu können.

## Klinisch wichtige prognostische Faktoren für das FTC

Wie oben ausgeführt spielt der histologische Subtyp eine Rolle für die Gesamtprognose [4, 31]. Wie bereits aus der WHO-Klassifikation hervorgeht, ist das Vorhandensein einer Angioinvasion prognostisch relevant. Hier hat sich eine Zahl von ≥ 4 Angioinvasionen als prognostisch bedeutsam gezeigt [14]. Es gibt jedoch in den letzten Jahren zahlreiche Arbeiten, in denen versucht wird, diesen Cut-off-Wert im Zusammenhang mit der Prognose zu optimieren. Diese haben jedoch bis zuletzt nicht zu einer Veränderung der Betrachtungsweise geführt, auch wenn zuletzt vorgeschlagen wurde, dass der Cut-off-Wert 2 Angioinvasionen relevanter sei [31].

In Bezug auf molekulare Veränderungen wurde in einer Studie gezeigt, dass der Nachweis von TERT-Promoter-Mutationen ein wichtiger Faktor für die Einschätzung der Prognose ist, da TERT-Promoter-Mutationen über alle Subtypen des FTC hinweg mit signifikant (*p* < 0,001) schlechteren Überlebensraten assoziiert waren [22].

Die Tumorgröße ist ein unabhängiger Risikofaktor für das Auftreten von Rezidiven

In Bezug auf die Tumorgröße ist beim FTC ein Tumor von mehr als 4 cm mit einer schlechteren Prognose assoziiert und wurde als unabhängiger Risikofaktor für das Auftreten von Rezidiven identifiziert (Odds Ratio = 6,750; Konfidenzintervall[CI]: 1,01–44,92; *p* < 0,048; [23]). Dieser Zusammenhang wurde in einer Metaanalyse aus 13 Studien mit 2075 Patienten bestätigt [32]. Hierbei scheint nicht nur der Cut-off-Wert von 4 cm entscheidend, sondern die mit zunehmender Größe erhöhte Wahrscheinlichkeit des Auftretens zusätzlicher nachteiliger prognostischer Faktoren. Hierzu zählt das Vorhandensein einer extrathyroidalen Ausbreitung, das relevant für das Auftreten von Rezidiven und das Gesamtüberleben ist [11, 32].

Besonders die Fernmetastasierung ist ein wichtiger negativer prognostischer Faktor. FTC metastasieren in erster Linie in Lunge und Knochen, es sind aber auch metastatische Absiedlungen in Leber, Gehirn und andere Organe beschrieben [18]. In minimal-invasiven FTC liegt die Häufigkeit der Metastasierung bei Erstdiagnose im Bereich von 1–9 %, während das breitinvasive FTC bei Erstdiagnose Metastasen in 8–45 % zeigen. Im Gegensatz zum PTC spielen Lokalrezidive beim FTC eine untergeordnete Rolle, sodass die Kontrolle einer möglichen Fernmetastasierung ein wichtiger Faktor für das Überleben von Patienten mit FTC ist [31].

Das Alter stellt einen weiteren Risikofaktor dar. Insgesamt ist ein höheres Lebensalter mit einer schlechteren Prognose in Bezug auf das krankheitsfreie Überleben und das Vorhandensein von Fernmetastasierung assoziiert. Ob ein Cut-off-Wert von 45 oder von 55 Jahren relevanter ist, kann für das FTC aufgrund heterogener Datenlage noch nicht abschließend nicht beurteilt werden [31].

Die vorgenannten Risikofaktoren höheres Lebensalter, Tumoren der Kategorie T3/4 (also > 4 cm), breitinvasives Wachstum, N1- und M1-Situation erhöhen auch die Wahrscheinlichkeit eines radiojodrefraktären Verlaufs, was ein aggressiveres chirurgisches Vorgehen rechtfertigt [26].

## Vorgeschlagene chirurgische Vorgehensweise beim FTC

Grundsätzlich orientiert sich das Resektionsausmaß an prognostisch relevanten Faktoren. Entsprechend bleibt bei den benignen follikulären Veränderungen und den benignen Neoplasien wie der NIFTP die Hemithyreoidektomie im Sinne eines diagnostisch durchgeführten Eingriffs ausreichend ([12]; Abb. 2).

### Merke.

*Vorgeschlagene chirurgische Vorgehensweise: Bei benignen follikulären Veränderungen und benignen Neoplasien ist die Hemithyreoidektomie im Sinne eines diagnostischen Eingriffs ausreichend.*

In Bezug auf das die Frage zum Resektionsausmaß beim FTC folgerten Zhang et al. in ihrer Metaanalyse von 2023, dass weniger die Frage einer Hemithyreoidektomie vs. Thyreoidektomie entscheidend sei, sondern die Tatsache, dass der Tumor R0-reseziert ist [32]. Zu einem ähnlichen Ergebnis kommt eine Studie aus dem gleichen Jahr: In einer Kohorte von 6871 Patienten mit FTC wurde der Einfluss des Resektionsausmaßes in Bezug auf das Gesamtüberleben im Zusammenhang mit der Tumorgröße bis 4 cm analysiert. In dieser zeigt sich nach multivariater Adjustierung, dass es im 5‑Jahres-Follow-up keinen Unterschied macht, ob Patienten eine Hemithyreoidektomie oder Thyreoidektomie erhalten haben [16]. Vergleichbar ist dies mit einer weiteren Studie mit 16.057 Patienten, die allerdings alle differenzierten Schilddrüsenkarzinome einschließt [7]. Insgesamt greifen diese Studien sicherlich zu kurz, da jeweils sämtliche histologische Subtypen gemeinsam verglichen wurden und oben ausgeführte Risikofaktoren nicht mit einbezogen wurden. Zusätzlich bleibt die oben dargestellte neue Klassifikation der Schilddrüsentumoren von 2022 in diesen Arbeiten unberücksichtigt. Nach dieser handelt es sich beim minimal-invasiven FTC um einen Low-risk-Tumor, bei dem eine lokale Resektion ausreicht [4]. Entsprechend zeigt sich in der Literatur Einigkeit darüber, dass beim minimal-invasiven FTC ohne Angioinvasion die Hemithyreoidektomie ausreichend ist ([9, 10, 13, 31]; Abb. 2).

### Merke.

*Vorgeschlagene chirurgische Vorgehensweise: Beim minimal-invasiven FTC* *<* *4* *cm ohne Risikofaktoren ist die Hemithyreoidektomie ausreichend. Eine prophylaktische Lymphknotendissektion ist nicht erforderlich.*

Beim Vorliegen zusätzlicher prognostisch relevanter Faktoren wie einer Größe über 4 cm sollte eine Thyreoidektomie – auch im Rahmen einer Nachresektion – durchgeführt werden. Auch wenn es dazu keine systematischen Untersuchungen gibt, erscheint es im Individualfall gerechtfertigt auch bei kleineren Tumoren mit Vorhandensein zusätzlicher Risikofaktoren wie der minimalen extrathyreoidalen Extension oder dem Nachweis prognostisch ungünstiger Mutationen eine Thyreoidektomie durchzuführen. Dies ermöglicht dann auch die Durchführung einer Radiojodtherapie. Aufgrund der schlechteren Prognose und zur Verhinderung von Fernmetastasen sollte bei FTC mit Angioinvasion eine Thyreoidektomie mit anschließender Radiojodtherapie durchgeführt werden [4, 31].

### Merke.

*Vorgeschlagene chirurgische Vorgehensweise: Beim minimal-invasiven FTC mit Angioinvasion sollte eine Thyreoidektomie durchgeführt werden. Bei postoperativer Diagnose sollte eine Komplettierungsoperation durchgeführt werden.*

Eine prophylaktische Lymphknotendissektion ist beim minimal-invasiven FTC aufgrund der sehr niedrigen Rate an Lymphknotenmetastasierung nicht erforderlich [3]. Beim Vorhandensein von Lymphknotenmetastasen wird eine Kompartmentresektion empfohlen [13].

Beim breitinvasiven FTC ist die Durchführung einer Thyreoidektomie mit anschließender Radiojodtherapie aufgrund der insgesamt schlechteren Prognose und des aggressiveren Wachstumsverhaltens sinnvoll [4]. Das breitinvasive FTC lässt sich häufig bereits intraoperativ im Gefrierschnitt nachweisen. Im Gegensatz zum minimal-invasiven FTC ist beim breitinvasiven Subtyp die primäre Rate an Lymphknotenmetastasen mit 20 % hoch und in diesem Fall meist vergesellschaftet mit dem Vorhandensein von Fernmetastasen. Aus diesem Grund wurde für solche Fälle eine prophylaktische zentrale Lymphknotendissektion in der letzten deutschen Leitlinie vorgeschlagen [10]. Diese ist ansonsten nur bei Nachweis von Lymphknotenmetastasen indiziert [5, 13].

### Merke.

*Vorgeschlagene chirurgische Vorgehensweise: Beim breitinvasiven follikulären Schilddrüsenkarzinom (WIFTC) sollte eine Thyreoidektomie durchgeführt werden.*

Das onkozytäre Schilddrüsenkarzinom (OTC) ist, wie oben ausgeführt, durch eine insgesamt aggressivere Tumorbiologie gekennzeichnet. Dies rechtfertigt die Empfehlung einer Thyreoidektomie als Primär- oder Komplettierungsoperation bei postoperativer Diagnose. Wenn das OTC bereits im Rahmen der Primäroperation nachzuweisen ist, sollte in diesem Fall auch eine prophylaktische Lymphknotendissektion durchgeführt werden, dies vor allem deshalb, da beim OTC eine geringere Wirksamkeit der Radiojodtherapie angenommen wird. Beim Nachweis von Lymphknotenmetastasen sollte eine befallsorientierte Lymphknotendissektion durchgeführt werden sollte. Diese Vorschläge lehnen sich an die Empfehlungen zum vormaligen „Hürthle-Zell“-/oxyphilen Karzinom an [10].

### Merke.

*Vorgeschlagene chirurgische Vorgehensweise: Beim onkozytären Schilddrüsenkarzinom (OTC) sollte eine Thyreoidektomie durchgeführt werden. Beim Nachweis eines OTC prä- oder intraoperativ sollte eine prophylaktische zentrale Lymphknotendissektion durchgeführt werden*.

## Fazit für die Praxis

- Die dargestellte chirurgische Vorgehensweise basiert überwiegend auf älteren Arbeiten. Nach bisherigem Kenntnisstand fehlen neuere Daten, die eine tiefgreifende Veränderung des chirurgischen Vorgehens rechtfertigen.
- Es bleibt abzuwarten, ob die retrospektive Einordnung von Patientenkohorten nach der neuen WHO-Klassifikation hierfür neue Erkenntnisse bringen wird.
